# Sensitive and Rapid HPLC Method for Determination of Memantine in Human Plasma Using OPA Derivatization and Fluorescence Detection: Application to Pharmacokinetic Studies

**DOI:** 10.3797/scipharm.1008-17

**Published:** 2010-10-05

**Authors:** Afshin Zarghi, Alireza Shafaati, Seyed Mohsen Foroutan, Arash Khoddam, Babak Madadian

**Affiliations:** 1 Department of Pharmaceutical Chemistry, School of Pharmacy, Shahid Beheshti University of Medical Sciences, Tehran, Iran; 2 Department of Pharmaceutics, School of Pharmacy, Shahid Beheshti University of Medical Sciences, Tehran, Iran; 3 Noor Research and Educational Institute, Tehran, Iran

**Keywords:** Memantine, Derivatization, HPLC, Monolithic column, Pharmacokinetic study

## Abstract

A rapid, sensitive and reproducible HPLC method was developed and validated for the analysis of memantine in human plasma after derivatization with *o*-phthaldialdehyde (OPA) and fluorescence detection. Amantadine was used as internal standard. The derivatized memantine and amantadine were eluted in less than 10 min with no interference from endogenous plasma peaks. The analysis was carried out on a monolithic silica column (Chromolith Performance RP-18e, 100×4.6 mm). The mobile phase was composed of a mixture of acetonitrile and 0.025 M phosphate buffer (50:50, v/v, pH=4.6) with a flow rate of 2.5 mLmin^−1^. The excitation and emission wavelengths were set at 335 nm and 440 nm respectively. The assay enables the measurement of memantine for therapeutic drug monitoring with a lower quantification limit of 2 ngmL^−1^. The method involves simple extraction procedure and analytical recovery was 82.8± 0.9%. The calibration curve was linear over the concentration range 2–80 ngmL^−1^. The coefficients of variation for inter-day and intra-day assay were found to be less than 8%. The method was successfully applied to pharmacokinetic studies in humans.

## Introduction

**Fig. 1.**
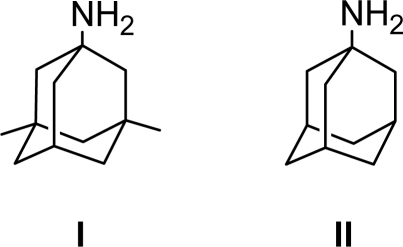
Chemical structures of memantine **I** and amantadine **II**

Memantine (**I**, Fig. 1), 1-amino-3,5-dimethyladamantane hydrochloride, is an adamantine derivative administered orally for many neurologic disorders, including Alzheimer’s disease and Parkinson [1–4]. It has been used in other disorders such as brain injury or comatose state. Memantine is readily absorbed from the gastro-intestinal tract with peak concentrations in plasma occurring ranges from 3 to 8 hours after administration by mouth. It is poorly metabolized by the liver and about 70% of the administered dose excreted, unchanged, in the urine. There have been some reports about the analysis of memantine determination by HPLC [5, 6], GC-MS [7, 8], and LC-MS [9–11]. However, some of these methods were not developed to determine memantine in plasma samples because the interfering endogenous substances in biological samples made the analysis more complex than those in preparations [5, 6], while others have either high limit of quantification (LOQ) or are too much complex, which limit their application for a large number of samples. Additionally, for the sample preparation, most of these methods require tedious extraction procedures, which are time-consuming, complex or both [6–10]. Moreover, some of aforementioned methods need long chromatographic elution time for analysis of memantine in plasma and were not suitable in all conditions [5, 6]. A GC/MS method has been also reported for determination of memantine in plasma by Kornhuber et al [8]. However, the method had low sensitivity (LOQ= 5 ngmL^−1^) compared with LC-fluorescence and LC-MS methods and therefore is not suitable for pharmacokinetic studies properties. LC methods based on MS or MS-MS as the detection system for the analysis of memantine in plasma are very sensitive, having low quantitation limits. However, these methods are not available for most laboratories because of their specialty requirement and financial reasons. The present study describes a rapid and sensitive HPLC method based on derivatization with *o*-phthaldialdehyde (OPA) with fluorescence detection, which enables the determination of memantine with good accuracy at low drug concentrations in plasma using simple extraction procedure. Separation was performed on a reversed-phase monolithic column, which has lower separation impedance comparing to the particulate packings, and therefore it allows easy optimizing chromatographic conditions to obtain desirable resolution in a short time. The sample preparation only involves a simple extraction procedure and no evaporation step is required. We also demonstrate the applicability of this method for pharmacokinetic studies in humans.

## Material and methods

### 1. Chemicals

Memantine and amantadine (**II)** were supplied by Osveh Pharmaceuticals (Tehran, Iran). Memantine is available as oral tablet containing 10 mg of memantine and other inactive ingredients. HPLC-grade acetonitrile and all other chemicals were obtained from Merck (Darmstadt, Germany). Water was obtained by double distillation and purified additionally with a Milli-Q system.

### 2. Instruments and chromatographic conditions

The chromatographic apparatus consisted of a model Wellchrom K-1001 pump, a model Rheodyne 7125 injector and a model RX-10AXL fluorescence detector connected to a model Eurochrom 2000 integrator, all from Knauer (Berlin, Germany). The separation was performed on Chromolith Performance (RP-18e, 100×4.6 mm) column from Merck (Darmstadt, Germany). The mobile phase consisted acetonitrile and 0.025 M phosphate buffer (50:50, v/v) adjusted at pH=4.6 with a flow rate of 2.5 mLmin^−1^. The excitation and emission wavelengths were set at 335 nm and 440 nm respectively. The mobile phase was prepared daily and degassed by ultrasonication before use. The mobile phase was not allowed to recirculate during the analysis.

### 3. Standard solutions

Stock solutions (1 mgmL^−1^ as the free base) of memantine hydrochloride was prepared in 0.1 M HCl. Then 20, 100, 200, 400, 600 and 800 ngmL^−1^ working standards were prepared in 0.01 M HCl and stored at +4 °C.

The solution of amantadine hydrochloride, internal standard was prepared by dissolving 10 mg amantadine hydrochloride in 10 mL 0.1 M HCl to obtain a concentration of 1 mgmL^−1^. The final solution was obtained by diluting this solution with 0.01M HCl to give concentration of 500 ngmL^−1^ of amantadine hydrochloride and stored at +4 °C.

### 4. Sample preparation

To 430 μL of plasma in a glass-stoppered 15 mL centrifuge tube were added 20 μL of amantadine as internal standard (500 ngmL^−1^), 50 μL of 2.5 M NaOH solution and 100 mg NaCl. Then, the mixture was vortexed for 1 min and extracted with 750 μL of *n*-hexane. After mixing (30 s), the mixture centrifuged for 5 min at 8000 rpm. Then, 600 μL of organic phase was transferred to small-volume tube and 50 μL of 0.01 M HCl was added and vortexed for 1 min. The top organic layer was aspirated and discarded. To aqueous acid phase, 100 μL of borate buffer (0.1 M, pH= 9.5) and 200 μL of OPA derivatization solution (1 mL of 0.3 M OPA in methanol, 50μ μL of mercaptoethanol and 10 mL of 0.1 M borate buffer, pH= 9.5) were added and vortexed. The mixture was allowed to react for 1 min at room temperature. Then, 100 μL acetonitrile and 100 mg NaCl were added. The mixture was centrifuged s for 10 min at 8000 rpm and 30 μL of supernatant was injected into liquid chromatograph.

### 5. Biological samples

24 male healthy volunteers were included in this study. The study protocol was approved by the Ethics Committee of Shahid Beheshti University of Medical Sciences and written informed consent was obtained from the volunteers. Memantine was administered in a single dose of 20 mg to the volunteers after overnight fasting. Plasma samples were collected at 1, 2, 2.5, 3, 3.5, 4, 5, 6, 7, 8,10, 24, 48 and 72 h after dosing and then frozen immediately at −20 °C until assayed.

### 6. Stability

The stability of memantine was assessed for spiked plasma samples stored at −20 °C for up to 6 months and at ambient temperature for at least 24 h. The stability of stock solutions stored at −20 °C was determined for up to one month by injecting appropriate dilutions of stocks in 0.01 HCl on day 1, 15 and 30 and comparing their peak areas with fresh stock prepared on the day of analysis. Samples were considered to be stable, if the assay values were within the acceptable limits of accuracy and precision.

### 7. Plasma standard curve

Blank plasma was prepared from heparinized whole-blood samples collected from healthy volunteers and stored at −20 °C. After thawing, 50 μL of one of the above-mentioned memantine working standards were added to yield final concentrations of 2, 10, 20, 40, 60 and 80 ngmL^−1^. Internal standard solution was added to each of these samples to yield a concentration of 50 ngmL^−1^. The samples were then prepared for analysis as described above.

Calibration curves were constructed by plotting peak area ratio (y) of memantine to the internal standard versus memantine concentrations (x). A linear regression was used for quantitation.

### 8. Precision and accuracy

The precision and accuracy of the method were examined by adding known amounts of memantine to pool plasma (quality control samples). For intra-day precision and accuracy six replicate quality control samples at each concentration were assayed on the same day. The inter-day precision and accuracy were evaluated on three different days.

### 9. Limit of quantification (LOQ) and recovery

For the concentration to be accepted as LOQ, the percent deviation from the nominal concentration (accuracy) and the relative standard deviation must be ±10% and less than 10%, respectively, considering at least six-times the response compared to the blank response. The analytical recovery for plasma at four different concentrations of memantine (5, 20 and 60 ngmL^−1^) was determined. Known amounts of memantine were added to drug-free plasma and the internal standard was then added. The relative recovery of memantine was calculated by comparing the peak areas for extracted memantine from spiked plasma and a standard solution of memantine in 0.01 M HCl containing internal standard with the same initial concentration (six samples for each concentration level).

### 10. Selectivity and specificity

Control human plasma, obtained from twelve healthy volunteers, was assessed by the procedure as described above and compared with respective plasma samples to evaluate selectivity of the method. Some antidepressant and antipsychotic drugs were tested for potential interferences [6].

### 11. Pharmacokinetic Analysis

Memantine pharmacokinetic parameters were determined by non compartmental methods. Elimination rate constant (K) were estimated by the least-square regression of plasma concentration-time data points lying in the terminal log-linear region of the curves. Half-life (T_1/2_) was calculated as 0.693 divided by K. The area under the plasma concentration-time curve from time zero to the last measurable concentration at time t (AUC_0-t_) was calculated using the trapezoidal rule. The area was extrapolated to infinity (AUC_0-∞_) by addition of C_t_/K to AUC_0-t_ where C_t_ is the last detectable drug concentration. Peak plasma concentration (C_max_) and time to peak concentration (T_max_) were derived from the individual subject concentration- time curves.

## Results and Discussion

**Fig. 2.**
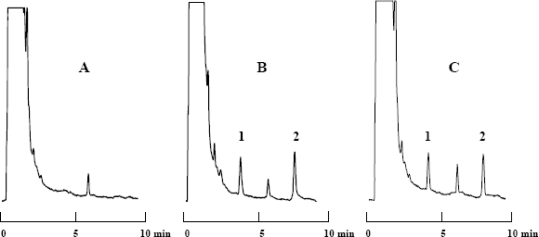
Chromatograms of (A) blank plasma; (B) blank plasma spiked with 30 ngmL^−1^ memantine (1) and 50 ngmL^−1^ amantadine (2, internal standard); (C) plasma sample from a healthy volunteer 3 h after oral administration 20 mg memantine.

Under the chromatographic conditions described, memantine and the internal standard peaks were well resolved. Endogenous plasma components did not give any interfering peaks. Separation was performed on a reversed-phase monolithic column, which has lower separation impedance comparing to the particulate packings, and therefore it allows easy optimizing chromatographic conditions to obtain desirable resolution in a short time. Accordingly, the chromatographic elution step is undertaken in a short time (∼ 8 min) with high resolution. Figure 2 shows typical chromatograms of blank plasma in comparison to spiked samples analyzed for a pharmacokinetic study. The average retention times of memantine and amantadine were 4.2 and 8.1 min, respectively. The formation of the OPA/thiol derivatives of memantine and amantadine is simple and rapid. Reactions proceed to completion at room temperature in 1 min using mercaptoethanol as the reducing agent. The derivatization reaction depends on pH of reaction medium and it should be kept above pH 9.0. The repeatability of derivatization reaction was evaluated by relative standard deviation (RSD) of six repetitive injections of working standard solutions. The RSD of peak area repeatability of memantine was less than 0.5%. The derivatized memantine was stable enough so that re-injection of the extract the following day was possible using the same calibration curve. Re-injection of the memantine calibration standards 12 h following derivatization did not show any deterioration in peak area ratios (RSD < 1%) or overall sensitivity. The excitation and emission wavelengths for the detection of derivatized memantine and amantadine were optimized to obtain the greatest sensitivity under chromatographic conditions described.

To investigate possible interference from commonly co-administered drugs, several antipsychotic and antidepressant drugs such as olanzepine, risperidone, clozapine, citalopram, fluvoxamine, fluoxetine, sertaline and bupropin were analysed under our chromatographic condition. None of the drugs mentioned above interfered with analytes peaks as well. Most of these drugs did not derivatize or elute as solvent front. For the sample preparation, several tedious extraction methods have been used for analysis of memantine in biological fluids [5–9]. In addition, some of the methods used large volumes of plasma samples or toxic extraction agents which limiting their application to analyze large numbers of clinical samples. In our method, sample preparation involves simple extraction procedure and no evaporation step is required. The analytical recovery for plasma at three different concentrations of memantine was determined. Known amounts of memantine were added to drug-free plasma in concentrations ranging from 5–60 ngmL^−1^. The internal standard was added and the absolute recovery of memantine was calculated by comparing the peak areas for extracted memantine from spiked plasma and a standard solution of memantine containing internal standard with the same initial concentration. As shown in Table 1. the average recovery of memantine, determined at three different concentrations (5, 30 and 60 ngmL^−1^), was 83.2± 0.9% (n=6). The calibration curve for the determination of memantine in plasma was linear over the range 2–80 ngmL^−1^. The linearity of this method was statistically confirmed. For each calibration curve, the intercept was not statistically different from zero. The correlation coefficients (r) for calibration curves were equal to or better than 0.995. The slops of plasma standard curves in the nine different preparations were practically the same (the CVs were less than 2% for the slops of plasma standard curves). For each point of calibration standards, the concentrations were recalculated from the equation of the linear regression curves (Table 2). Using OPA dervatization and fluorescence detection, the limit of quantification (LOQ), as previously defined, was 2 ngmL^−1^ for memantine, which was better than LOQ reported by GC-MS assay [7, 8] or the HPLC-fluorescence assay dervatized with dansyl chloride [6]. This is sensitive enough for drug monitoring and other purposes such as pharmacokinetic studies.

We assessed the precision of the method by repeated analysis of plasma specimens containing known concentrations of memantine. As shown in Table 3, coefficients of variation were less than 8%, which is acceptable for the routine measurement of memantine. Stability was determined for spiked plasma samples under the conditions as previously described. The results showed that the samples were stable during the mentioned conditions. The aim of our study was to develop a rapid and sensitive method for the routine analysis of biological samples in pharmacokinetic memantine research. This method is well suited for routine application in the clinical laboratory because of the speed of analysis and simple extraction procedure.

**Fig. 3.**
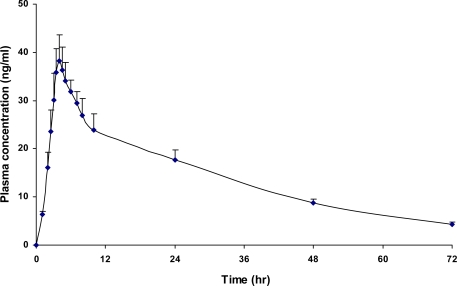
Mean plasma concentration-time profile of memantine in healthy volunteers (n=24) after a single 20 mg memantine.

Over 700 plasma samples were analyzed by this method without any significant loss of resolution. No change in the column efficiency and back pressure was also observed over the entire study time, thus proving its suitability. In this study plasma concentrations were determined in 24 healthy volunteers, who received 20 mg of memantine each. Fig 3. shows the mean plasma concentration-time profile of memantine. The derived pharmacokinetic parameters of 24 healthy volunteers are summarized in Table 4.

## Conclusions

A rapid and sensitive HPLC method has been described for analysis of memantine in plasma. Derivatization with OPA is very effective method for enhancing the chromatographic detection of memantine and other structurally related compound such as amantadine. Using monolithic column, the chromatographic elution step is undertaken in a short time with high resolution. In addition, the use of a simple sample preparation procedure instead of more complex extraction procedures makes this method suitable for pharmacokinetic and bioequivalence studies of memantine in humans.

## Figures and Tables

**Tab. 1.** Recovery data of memantine from plasma

**Memantine spiked concentration (ngmL^−1^)**	**Memantine concentration found (n=6)**	**Recovery (mean ±SD)%**
5	4.1	82.0±1.1
30	25.2	84.0±0.9
60	50.1	83.5±0.8

**Tab. 2.** Assay linearity

**Coefficient of the linear Regression analysis (r±SD)**		**Slope±SD**	**Intercept±SD**
Intra-assay	0.9968±5.60×10^−4^	0.0375±0.0005	0.061±0.003
n= 6	RSD= 0.056%	RSD= 1.33%	
Inter-assay	0.9955±6.20×10^−4^	0.0381±0.0006	0.063±0.004
n= 9	RSD= 0.062%	RSD= 1.57%	

**Tab. 3.** Reproducibility of the analysis of memantine in human plasma (n= 6)

**Concentration added (ngmL^−1^)**	**Concentration measured (mean ±S.E.)**	
	**Intra-day**	**Inter-day**
5	5.21 ± 0.39 (7.5)	4.81 ± 0.68 (7.1)
30	29.62 ± 2.14 (7.2)	27.76 ± 1.72 (6.2)
60	61.89 ± 2.91 (4.7)	57.36 ± 0.11 (5.4)

Values in parentheses are coefficients of variation (%).

**Tab. 4.** Pharmacokinetic parameters of memantine in healthy volunteers (n=24) following a single oral dose of 20 mg of memantine

**Parameter**	**Result (mean±SD)**
T_max_ (h)	3.72±0.40
C_max_ (ngmL^−1^)	39.51±8.72
AUC_0-t_ (ng.hmL^−1^)	1116.55±236.59
AUC_0-∞_ (ng.hmL^−1^)	1262.99±263.35
K_el_ (h^−1^)	0.029±0.001
T_1/2_ (h)	23.85±1.52
